# Dendrites of Neocortical Pyramidal Neurons: The Key to Understand Intellectual Disability

**DOI:** 10.1007/s10571-021-01123-1

**Published:** 2021-07-03

**Authors:** Alberto Granato, Adalberto Merighi

**Affiliations:** Department of Veterinary Sciences, University of Turin, Largo Paolo Braccini 2, 10095 Grugliasco, TO Italy

**Keywords:** Cerebral cortex, Down syndrome, Fetal alcohol, Calcium, Dendritic spike, Apical dendrite

## Abstract

Pyramidal neurons (PNs) are the most abundant cells of the neocortex and display a vast dendritic tree, divided into basal and apical compartments. Morphological and functional anomalies of PN dendrites are at the basis of virtually all neurological and mental disorders, including intellectual disability. Here, we provide evidence that the cognitive deficits observed in different types of intellectual disability might be sustained by different parts of the PN dendritic tree, or by a dysregulation of their interaction.

The father of modern Neuroscience, Santiago Ramón y Cajal, postulated that neocortical pyramidal neurons (PNs) might play an outstanding role for the accomplishment of higher cognitive functions. He defined these cells, representing the vast majority of neocortical neurons, the “psychic cells” (Cajal 1917). Despite his feeling of shame, aimed at counterbalancing the “audacity of language,” after more than a century we have to recognize that, once again, the claim of the great Spanish scientist was substantially right. In recent years, it has been shown that PNs, despite their apparent morphological homogeneity, are specialized for different physiological/behavioral functions in different cortical areas and species (see, for review, Jacobs and Scheibel 2002; Elston 2003; Spruston 2008; Luebke 2017). Moreover, cortical areas composed of specialized pyramidal cells are characterized by unique connectivity and capacity, with size of the dendritic tree and number of spines increasing progressively from primary to higher order areas (Elston 2007). These regional specializations in pyramidal cell structure and circuit connectivity are important for hierarchical and/or distributed processing (Elston 2003, 2007; Elston and Fujita 2014). On the same line of reasoning, it can be assumed that a functional derangement of PNs is the pathophysiological basis of the cognitive deficit observed in intellectual disability (Granato and De Giorgio 2014).

Intellectual disability (ID), previously referred to as mental retardation, is classically defined as a neurodevelopmental disorder with IQ below 70, although more complex definitions, based on poor adaptive functioning and reduced daily life skills, have been provided by the DSM-5 (American Psychiatric Association 2013) and the American Association on Intellectual Developmental Disabilities (Shogren and Turnbull 2010).

The present point of view deals with the anomalies of neocortical PNs, as observed in experimental studies reproducing known causes of ID, as well as in the brains of affected human individuals. Given that dendritic alterations are considered among the most relevant anatomical and functional counterparts of ID (Kaufmann and Moser 2000) and owing to the great extension and geometric complexity of the dendritic arborizations of PNs, we shall focus primarily on dendritic anomalies. Some excellent reviews cover exhaustively the relationships between dendritic alterations and ID (Kaufmann and Moser 2000; Dierssen and Ramakers 2006; Quach et al. 2021). Our purpose is to provide mechanistic insights into how the disruption of PN dendritic function contributes to the genesis of ID, with a special emphasis on the role of the different parts of the dendritic tree.

## Dendrites of PNs

**Fig. 1:**
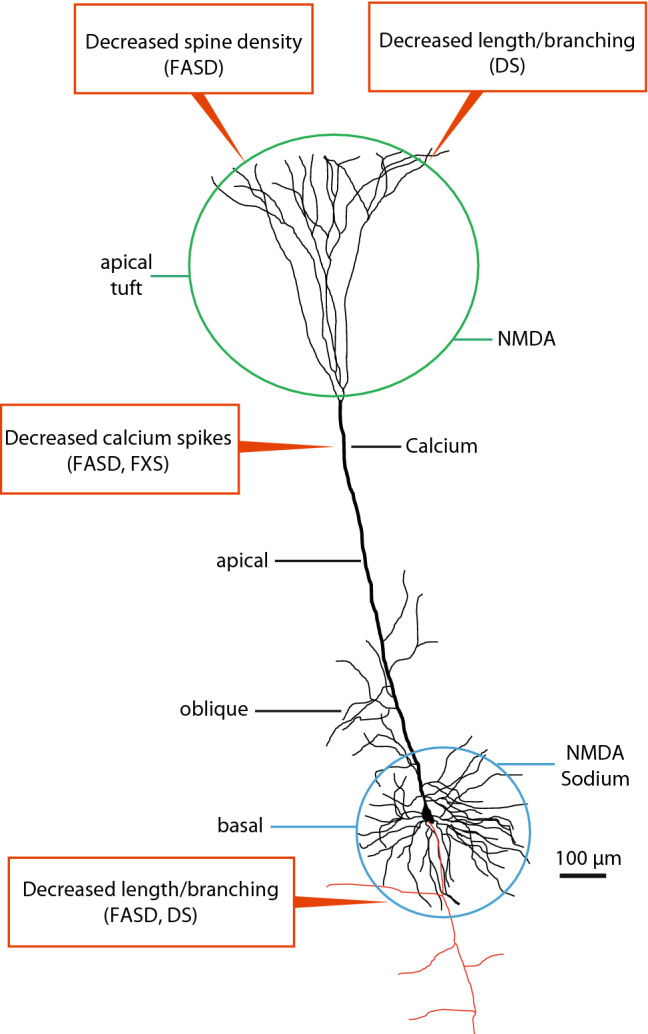
3D reconstruction of a thick-tufted PN of layer 5. On the left of the neuron there is the indication of the different sections of the dendritic tree. On the right the main regenerative events occurring in that dendritic domain, according to Larkum et al. 2009. Red: axon. The text boxes indicate some of the alterations occurring in FASD, FXS, and DS, along with the dendritic domain affected (see the text for further explanation)

PNs represent the majority of neocortical neurons and are distributed in all cortical layers except layer 1. Among PNs, the thick-tufted cells are the most thoroughly studied, provide the cortical output directed to subcortical structures, and can be found in the deep part of layer 5 [layer 5b (Spruston 2008; Ramaswamy and Markram 2015)]. Thick-tufted PNs are characterized by a prominent apical dendrite spanning all the way to the pial surface and terminating with a branching apical tuft, whose radium often equals (or exceeds) that of the basal dendrites (Fig. 1). Other classes of PNs are those of layer 2/3 (providing cortico-cortical associative and callosal projections) and those bearing a slender apical dendrite, mainly located in the superficial part of layer 5 (Krieger et al. 2017; see also Fig. 1 in Shepherd 2013). Modified PNs projecting to the thalamus and claustrum reside in layer 6 (Thomson 2010).

The prototypical PNs, the thick-tufted cells of layer 5, display geometric differences among the basal, oblique, and apical dendritic domains that are clearly recognizable at a first glance (see Fig. 1). In a seminal paper published exactly thirty years ago, Alan Larkman provided a rigorous formal description of such branching pattern differences, pointing out, for instance, that basal dendrites branching points are close to the cell body, whereas intermediate branches of the apical tuft are relatively longer than distal ones (Larkman 1991). It is well known that the dendritic geometry impinges upon the functional properties of neurons (Mainen and Sejnowski 1996). Furthermore, different parts of the dendritic tree of layer 5 PNs are potentially involved in the microcircuitry of different cortical layers. Therefore, it is conceivable that basal and apical dendrites contribute differently to the cortical machinery during physiological cognitive tasks.

Regarding the connections, the basal dendrites receive feedforward input from the receptor surfaces through the thalamic relay. The ascending input is thought to be conveyed indirectly to the dendrites of layer 5 through the canonical cortical circuit [thalamus → layer 4 → layer 2/3 → layer 5 (Thomson and Morris 2002; Lübke and Feldmeyer 2007)]. However, layer 5 neurons can be also contacted directly by thalamic afferents (Meyer et al. 2010; Constantinople and Bruno 2013). Conversely, most of the apical tuft of PNs lies in layer 1, where it receives feedback connections from higher cortical areas, conveying input related to attention, context, and expectations (Coogan and Burkhalter 1990; Cauller 1995; Cauller et al. 1998). Together, the basal and apical dendritic arborization are in the ideal position to integrate bottom-up and top-down streams of information. The refinement of dendritic recording (Davie et al. 2006) made it possible to ascertain that action potentials can backpropagate through the apical dendrite of PNs (Stuart and Sakmann 1994) and to shed light on the interplay between basal and apical dendrites. Using multiple patch-clamp recordings from the soma and the apical dendrite of layer 5 PNs, it has been demonstrated that the coincidence of a backpropagated action potential generated at the soma and of an apical dendritic input is able to generate a dendritic calcium spike that, in turn, elicits a burst of somatic spikes (Larkum et al. 1999). This mechanism, originally called backpropagation-activated calcium spike firing (BAC firing) has been considered the electrophysiological basis of the top-down/bottom-up integration operated by a single PN (Larkum 2013). This idea was widened by Bill Phillips and Matthew Larkum, leading to the concept of “apical amplification,” the mean by which the information coming from the external world (bottom-up) is modulated by context-sensitive (top-down) information (Phillips et al. 2016; Phillips 2017). A role of the apical dendrite in cognition and consciousness has been also postulated by LaBerge (2006). Recently, it has been demonstrated that apical dendritic potentials can gate sensory perception and that such a modulation depends on contextual information (Takahashi et al. 2020). A dysregulation of context-modulated sensory perception and learning abilities can represent a prominent feature of ID (Alevriadou et al. 2004; Carr et al. 2010; Murray et al. 2019).

As to the distribution of ion channels on the membrane of different dendritic compartments, it has been proposed that the apical tuft and basal dendrites are dominated by NMDA receptors and associated potentials, while a calcium initiation zone, located just beneath the apical tuft, is endowed with voltage gated calcium channels (Nevian et al. 2007; Larkum et al. 2009); Fig. 1). Moreover, the correct degree of coupling between basal and apical compartments would be ensured by hyperpolarization-activated HCN channels, responsible for the Ih current and densely distributed on the apical dendrite of PNs (Nolan et al. 2004; Phillips et al. 2016). Interestingly, these ion channels are developmentally regulated (Atkinson and Williams 2009) and their dysregulation might be involved in the genesis of neurodevelopmental disorders (see below).

In the last years, a conspicuous line of research focused on the dual basal/apical organization of PNs. Changes in the apical amplification process have been implicated in the pathophysiology of several mental disorders, including schizophrenia (Phillips et al. 2016; Mäki-Marttunen et al. 2019). Furthermore, the integration of two different compartments with feedforward and feedback input seems to be ideally suited to bridge the gap between artificial intelligence and neuroscience, since there are similarities between deep learning algorithms and the functional subdivisions observed in PNs (Guerguiev et al. 2017).

Dendrites of pyramidal neurons are covered with spines, which receive most of synaptic inputs and are thought to play a central role in several functions, from electrical filter/isolation to synaptic and structural plasticity (reviewed in Yuste 2011; Sala and Segal 2014). The density of dendritic spines, as well as their pattern of developmental growth and reshaping, display significant differences in different cortical areas (Elston and Defelipe 2002; Elston and Fujita 2014). Moreover, the density and distribution of dendritic spines appear to be differently regulated in apical and basal dendrites of PNs during learning (Knafo et al. 2001), in response to hormones (Gould et al. 1990), and in experimental models of neurological illness (Perez-Cruz et al. 2011).

## PN Dendritic Domains and ID

From the features outlined above, it is clear that the extensive dendrites of neocortical PNs play a pivotal role in neural computation and higher functions. Therefore, the changes of PN dendrites appear to be central in the genesis of ID. Moreover, a disruption of the interplay between functionally distinct basal and apical compartments might contribute to the pathophysiology of several mental disorders, including ID. Here, we focus on PN dendritic alterations in some of the most frequently observed genetic and non-genetic types of ID. Among genetically determined IDs, Down syndrome (DS), caused by trisomy of the human chromosome 21, besides representing the most commonly identified form (Sherman et al. 2007), can be also reproduced by murine models (Dierssen et al. 2001). Notably, dendritic alterations have been reported in both species (reviewed in Benavides-Piccione et al. 2004). Interestingly, when compared to matched-age controls, PNs in the visual cortex of individuals with DS showed a higher complexity of dendritic branching during the first six months of postnatal life, followed by a reduction of branches thereafter, and dendritic alterations were evident both in the apical and basal dendrites (Becker et al. 1986). In addition to these alterations in branching, a reduction of spine density has been reported in the apical dendrite of humans affected by DS (Suetsugu and Mehraein 1980). It should be noted, however, that PNs of the prefrontal cortex in a murine model of DS display an increased density of spines (Thomazeau et al. 2014). An augmented number of dysmorphogenetic dendritic spines is a consistent feature of a common inherited cause of ID, the fragile X syndrome (FXS), characterized by mutations of the FMRP, the protein encoded by the *FMR1* gene (Bagni and Greenough 2005).

The most common form of non-genetic ID is the consequence of the exposure to alcohol in utero and is nowadays referred to as fetal alcohol spectrum disorders (FASD). Rodent experimental models of FASD allowed to clarify several aspects in the pathogenesis of ID, including those related to PN dendritic anomalies (Valenzuela et al. 2012). Experimental FASD might represent an interesting case of dissociation between dendritic anomalies of basal and apical compartments: after exposure to ethanol during the first week of postnatal life in rats, corresponding to the third trimester of gestation in humans, the basal dendritic branches of PNs are strongly simplified, both in the somatosensory and in the prefrontal cortex (Granato et al. 2003, 2012; Hamilton et al. 2010). Conversely, the spine density of layer 2/3 basal dendrites is not affected (Hamilton et al. 2010; De Giorgio and Granato 2015). Using the same experimental protocols, a specular alteration was observed in the apical dendrites, that featured normal branching properties along with a decreased spine density (Whitcher and Klintsova 2008; Granato et al. 2012; De Giorgio and Granato 2015). It is worth mentioning, however, that in other types of ID, such as congenital/neonatal hypothyroidism, the apical dendrite shows a modified branching pattern (Ipiña and Ruiz-Marcos 1986).

## PN Dendrite Physiology and ID

As noted above, active currents generated locally in the dendritic tree can play a fundamental role for the function of PNs. Disruption of dendritic potentials can be the signature of many neurological and mental disorders (Palmer 2014). In the experimental model of FASD, we have demonstrated that the generation of Ca^2+^ spikes in the apical dendrites of layer 5 PNs is strongly impaired (Granato et al. 2012). A derangement of dendritic Ca^2+^ signaling has been also reported in the murine model of FXS (Meredith et al. 2007) and might be part of a more complex set of dendritic channelopathies observed in this condition (Brager and Johnston 2014). Calcium spikes are required to induce synaptic plasticity (Kampa et al. 2006; Cichon and Gan 2015). In addition, they support the apical amplification mechanism described above, that in turn is thought to provide the neurobiological basis for context-sensitive perception and learning (Phillips 2017). Interestingly, shutting down the Ube3a protein leads to a selective defect of growth of PN apical dendrites (Miao et al. 2013). The deficiency of the Ube3a protein in humans is associated to the Angelman syndrome, a condition characterized by ID and whose murine model displays a deficit of contextual learning (Jiang et al. 1998). Besides a direct impairment of dendritic calcium electrogenesis, other factors can contribute to the anomalous function of the apical dendrite and to the genesis of ID. For instance, HCN channels, responsible for the Ih current, play a role in the interaction between the basal and the apical dendrites of PNs and some of their variants can be associated to ID (Marini et al. 2018). Another factor ensuring the communication between different dendritic compartments is represented by the backpropagation of axon potential along the apical dendrite. Loss of sodium channels sustaining the backpropagation can also lead to ID (Spratt et al. 2019). Finally, PNs participate in a cortical microcircuit to which dendrite-targeting GABAergic interneurons provide a substantial contribution (Markram et al. 2004; Palmer et al. 2012; DeFelipe et al. 2013). Furthermore, top-down projections from higher cortical areas can engage in a disinhibitory circuit by contacting inhibitory neurons (chiefly VIP-calretinin cells) that, in turn, synapse onto other inhibitory cells, thus activating PN dendrites. Such a disinhibitory circuit might represent part of the neuronal basis for the apical amplification, since it is ideally suited to be involved in recalling past experiences and exploiting contextual cues (Pi et al. 2013; Karnani et al. 2014). Interestingly, an increase in the number of calretinin interneurons has been reported both in a model of FASD and in the Ts65Dn model of DS (Granato 2006; Pérez-Cremades et al. 2010).

The dendrites of each PN receive several thousand synapses. Therefore, although we focused on the morpho-functional dendritic alterations observed in the most representative ID syndromes, many rare mutations of synaptic proteins and ligand-gated ion channels can be responsible for anomalies of the dendritic machinery and can lead to ID (reviewed in Vieira et al. 2021). A striking example is represented by the mutations of the NMDA receptor subunits, whose consequence is represented either by loss or by gain of function, with possible excitotoxic mechanisms mediating the damage in the latter case (Lemke et al. 2016; Fry et al. 2018). NMDA mutations can also result in modified synaptic plasticity (Shin et al. 2020) and interference with dendritic growth (Sceniak et al. 2019).

## Concluding Remarks

Considering that PNs are the most abundant neuronal type of the cerebral cortex, and that they bear a large dendritic tree, it turns out that PN dendrites occupy a considerable part of neocortical volume. Therefore, the primary role played by PN dendrites in cortical computation and their involvement in ID are quite obvious. Even though each ID syndrome seems to be characterized by a specific type of dendritic alteration, times are not yet mature to classify ID according, for instance, to the different dendritic domain primarily altered, to the spine density, or to the specific interplay between inhibitory interneurons and PN dendrites. Although such a classification might prove useful to steer the clinical and therapeutic interventions, any effort in this direction appears to be challenging. In fact, ID is a permanent condition that is usually established early during neural development, often through intermediate phases showing transient features (see, for example, the dendritic hypertrophy observed in DS). Furthermore, some of the pathologic features might merely represent a byproduct, or a compensation attempt, of primary alterations. This might be the case for the increased number of potentially disinhibitory interneurons observed in DS and FASD, or the increased density of dendritic spines in DS and FXS.

Studies on PN dendrites in humans affected by ID are shadowed by technical limitations, in particular, by the capricious Golgi staining. The refinement of recording and staining techniques, along with the increased possibility of modeling neurons and their dendrites, can help to improve the results of human investigations (Elston et al. 2001; Benavides-Piccione et al. 2013; Goriounova et al. 2018). Furthermore, animal models of ID allow detailed in vitro and in vivo explorations of dendrite and spine anomalies. Therefore, the collaboration among clinical, computational, and experimental neuroscientists will warrant a bright future for the research on ID and dendrites.
